# Carrageenan-induced acute inflammation in the mouse air pouch synovial model. Role of tumour necrosis factor

**DOI:** 10.1080/09629359791901

**Published:** 1997-02

**Authors:** M. Romano, R. Faggioni, M. Sironi, S. Sacco, B. Echtenacher, E. Di Santo, M. Salmona, P. Ghezzi

**Affiliations:** 1 Istituto di Ricerche Farmacologiche “Mario Negri” Via Eritrea 62 Milan 20157 Italy; 2 Universitat Regensburg Institut of Pathology Regensburg Germany

## Abstract

We used the mouse air pouch model of inflammation to study the interaction between cytokines, prostaglandin E_2_ (PGE_2_) and cell migration during the various phases of acute local inflammation induced by carrageenan. In serum, the levels of interleukin 1 (IL-1), interleukin 6 (IL-6), tumour necrosis factor (TNF), serum amiloid-A (SAA) and Fe^++^ were never different from controls, indicating that no systemic inflammatory changes were induced. Locally the exudate volume and the number of leukocytes recruited into the pouch increased progressively until 7 days after carrageenan. The same was true for PGE_2_ production. We could not measure IL-1 but the production of IL-6 and TNF reached a maximum after 5-24 h then quickly decreased. Anti-TNF antibodies inhibited cell migration by 50% 24 h after treatment. Pretreatment with interleukin 10 (IL-10) inhibited TNF production almost completely and cell migration by 60%. Carrageenan-induced inflammation was modulated by anti-inflammatory drugs. Pretreatment with dexamethasone (DEX) or indomethacin (INDO) inhibited cell migration and reduced the concentration of TNF in the exudate. Production of PGE_2_ or vascular permeability did not correlate with the number of cells in the pouch. Local TNF seems to play an important role in this model, particularly for leukocyte migration in the first phase of the inflammatory process. In conclusion, the air pouch seems to be a good model for studying the regulation of the early events of local inflammation, particularly the role of cytokines and cell migration.


Mediators of Inflammation, 6, 32–38 (1997)

### Carrageenan-induced acute inflammation in the mouse air pouch synovial model. Role of tumour necrosis factor

M. Romano,[1,CA] R. Faggioni,[1] M. Sironi,[1] S. Sacco,[1] B. Echtenacher,[2] E. Di Santo,[1] M. Salmona[1] and P. Ghezzi[1]

[1]Istituto di Ricerche Farmacologiche "Mario Negri", Via Eritrea 62, 20157 Milan, Italy; and [2]Universitat Regensburg, Institut of Pathology, Regensburg, Germany

[CA]Corresponding Author
Fax: (+39) 2 3546277

**We used the mouse air pouch model of inflammation to study the interaction between cytokines, prostaglandin $E_2$ ($PGE_2$) and cell migration during the various phases of acute local inflammation induced by carrageenan. In serum, the levels of interleukin 1 (IL-1), interleukin 6 (IL-6), tumour necrosis factor (TNF), serum amiloid-A (SAA) and $Fe^{++}$ were never different from controls, indicating that no systemic inflammatory changes were induced. Locally the exudate volume and the number of leukocytes recruited into the pouch increased progressively until 7 days after carrageenan. The same was true for $PGE_2$ production. We could not measure IL-1 but the production of IL-6 and TNF reached a maximum after 5–24 h then quickly decreased. Anti-TNF antibodies inhibited cell migration by 50% 24 h after treatment. Pretreatment with interleukin 10 (IL-10) inhibited TNF production almost completely and cell migration by 60%. Carrageenan-induced inflammation was modulated by anti-inflammatory drugs. Pretreatment with dexamethasone (DEX) or indomethacin (INDO) inhibited cell migration and reduced the concentration of TNF in the exudate. Production of $PGE_2$ or vascular permeability did not correlate with the number of cells in the pouch. Local TNF seems to play an important role in this model, particularly for leukocyte migration in the first phase of the inflammatory process. In conclusion, the air pouch seems to be a good model for studying the regulation of the early events of local inflammation, particularly the role of cytokines and cell migration.**

**Key words:** Air pouch, Inflammation, Tumour necrosis factor

### Introduction

Many attempts have been made to correlate serum tumour necrosis factor (TNF) levels with the severity of different inflammatory diseases[1] but clinical studies[2] and experimental work[3] in various models of septic shock suggest that systemic levels of TNF are not a good predictor of the potential outcome of inflammation. However, in experiments of paw oedema induced by carrageenan injection, the local amount of TNF correlated with the swelling of the paw and it was therefore considered a good indication of the inflammatory status.[3]

On the other hand, TNF seems to be an important mediator in the pathogenesis of rheumatoid arthritis.[4,5] Joint extracts from arthritic rats had significantly higher TNF levels ($1054 \pm 147$ pg/g of tissue) than joint extracts of normal rats ($110 \pm 42$ pg/g of tissue).[6] Anti-TNF antibodies are protective in animal models of arthritis[7] and clinical studies have shown an effect of anti-TNF antibodies in patients with rheumatoid arthritis.[8] We established an *in vivo* model of acute local inflammation in the mouse. This air pouch model of synovial inflammation, described by Sedgwick and coworkers,[9] consists of a subcutaneous injection of air on the back of rodents. This procedure induces the proliferation of cells that stratify on the surface of the cavity to form a structure similar to the synovia after 6 days.[10] Injection of carrageenan induces inflammation and the pouch serves as a reservoir of cells and mediators that can be easily measured in the fluid that accumulates locally.

We investigated the production of TNF in relation to interleukin (IL)-1 and IL-6, which are reported to be regulated by TNF in synovial fluid and in the serum of patients with rheumatoid arthritis,[8] and in relation to leukocyte

© 1997 Rapid Science Publishers migration. Because prostaglandin $E_2$ ($PGE_2$) suppresses various leukocyte functions,[11,12] dose-dependently regulating TNF production by macrophages,[13] we also studied $PGE_2$ production in the air pouch.

We characterized the experimental model in terms of cytokine production as well as $PGE_2$ production, cell migration and vascular permeability (measured as extravasation of fluorescently labelled bovine serum albumin). The kinetics of all these parameters were investigated during the inflammatory response.

We also investigated whether a systemic acute phase response was induced. We measured serum TNF, IL-6 and IL-1 as well as serum amyloid A (SAA) and serum $Fe^{++}$ as typical markers of the acute phase response.[14] We used the cyclooxygenase inhibitor indomethacin (INDO) to investigate the role of $PGE_2$. We also used dexamethasone (DEX) and IL-10 potent inhibitors of the production of inflammatory cytokines that are protective in animal models of TNF-mediated endotoxic shock[15,16] and an anti-TNF antibody that neutralizes TNF *in vivo* and protects against endotoxic shock.[17] To evaluate the role of the polymorphonuclear leukocytes' (PMN) recruitment in the production of inflammatory mediators, we induced neutropenia by pretreating mice with 5-fluorouracil (5-FU).

### Materials and Methods

### Animals

Male CD1 mice, 22–25 g body weight (Charles River Italy, Calco, CO) were used for all experiments if not otherwise stated. The animals were housed at constant temperature ($20 \pm 1$°C) and relative humidity ($60 \pm 10$%) and provided with food and water *ad libitum*. All the procedures involving animals and their care were conducted in conformity with national and international laws and policies (EEC Council Directive 86609, OJ L358, 1, 12 December 1987; Italian Legislative Decree 116/92, Gazzetta Ufficiale della Repubblica Italiana n. 40, 18 February 1992; NIH Guide for the Care and the Use of Laboratory Animals, NIH publication no. 85–23, 1985).

### Air pouch model

After a period of adaptation the animals were anaesthetized with ether and 5 ml of air were injected under the skin on their back. After 3 days the pouches were reinjected with 3 ml of air. On day 6, 1 ml of 1% carrageenan (Sigma) in saline was injected into the pouch. The controls received 1 ml of saline. At different times after carrageenan the animals were anaesthetized and the pouches were washed with 1 ml of saline. The lavage fluid was immediately cooled on ice and the volume was recorded, then 50 µl were used for cell count after staining with erythrosin. The remaining fluid was centrifuged at 5000 rpm for 10 min at 4°C and supernatants were stored at −20°C until used for the measure of $PGE_2$, TNF or IL-6.

### Materials

IL-10 was a kind gift from Schering Plough, Italy. Dexamethasone phosphate was a kind gift from Laboratorio Farmacologico Milanese S.R.L. (Caronno P. Varese, Italy); indomethacin, water-soluble salt, was a commercial preparation from Chiesi Farmaceeutici (Parma, Italy). All drugs were given in saline. A rat anti-mouse TNF monoclonal antibody (V1q) was prepared and administered to mice as previously described.[18] The specificity and activity of this antibody has been described[19] and it protects mice against the lethality of LPS.[18,19]

### Mediator assays

TNF was measured by a standard cytotoxicity assay using L929 cells. IL-1 was measured using a mouse ELISA kit purchased from Genzyme (Cambridge, MA). IL-6 was measured as hybridoma growth factor on 7TD1 cell line. $PGE_2$ was measured with a commercial ELISA kit (Amersham, Little Chalfont, UK). Serum amyloid-A was determined using an ELISA as previously described.[20]

### Measurement of plasma exudation

Plasma exudation in the pouch was measured using fluorescein isothiocyanate-conjugated bovine serum albumin (FITC-BSA) (Sigma Chemical Co., St Louis, MO). At different times after carrageenan treatment, 0.6 mg/mouse of fluorescein isothiocyanate-conjugated bovine serum albumin dissolved in 0.2 ml of saline were injected in the tail vein of the animals; 30 min later the animals were anaesthetized with ether and the blood was collected. Fluorescence of the undiluted pouch fluid and of serum, diluted 1:10 in saline, were measured with the excitation wavelength set at 490 nm and emission at 521 nm. Plasma exudation was calculated as the fluorescence in the pouch as a percentage of that in serum.

### Statistical analysis

Data were analysed with one-way analysis of variance coupled with Duncan's test for multiple comparisons or with Kruskal–Wallis nonparametric test coupled with Sach's test for multiple comparisons.

### Results

### Characterization of the air pouch carrageenan inflammation model

Fig. 1 shows the kinetics of the volume of exudate and leukocyte recruitment during carrageenan-induced inflammation in the air pouch. The total number of leukocytes (Fig. 1A) was very similar in control and treated groups until 5 h after treatment. From 24 h to 7 days there was massive recruitment of cells into the pouches of animals treated with carrageenan. The cells were mostly PMN until 24 h after treatment and changed to a prevalence of macrophages from 48 hours on (data not shown). After 24 h PMN amounted to 70–80% of the total. Fig. 1B shows the volume of exudate recovered from the pouches after saline (control) or 1 ml of 1% carrageenan. In saline-treated animals the volume decreased due to the absorption of the saline injected at time 0. In carrageenan-treated animals the volume increased from 24 h to 7 days.

TNF and IL-6 concentrations (Fig. 2A and B) measured in the pouch, were undetectable in control animals at all times. In carrageenan-treated animals TNF and IL-6 both peaked 24 h after treatment, then declined very rapidly. Prostaglandin $E_2$ in pouch exudates (Fig. 3A) was never detectable in control animals but in carrageenan-treated animals there was a continuous increase from 5 h to 7 days. Plasma exudation measured as the percentage of FITC-BSA in the pouch compared with serum was considered as an indicator of the changes of vessel permeability induced by inflammation. Injection of carrageenan into the pouch significantly increased plasma exudation after 30 min, 5 h and 24 h (Fig. 3B). IL-1β was never found in pouch exudate (detection limit $\leqslant$ 50 pg/ml). To detect any systemic inflammatory effects after carrageenan in the air pouch, we measured circulating levels of IL-1β, IL-6, TNF and the parameters associated with an acute phase response: acute phase SAA and serum iron. We did not find hypoferraemia and TNF, IL-1, IL-6 and SAA were all below the limit of detection (data not shown), indicating that no systemic inflammatory effects were induced. To investigate whether the inflammation was confined to the periphery because of the structure of the pouch or the type of stimulus, we injected 100 ng/mouse of LPS into the pouch instead of carrageenan. In this case TNF, measured 90 min after treatment was increased both in pouch exudate (21399 ± 9389 pg/ml) and in serum (502 ± 173 pg/ml).

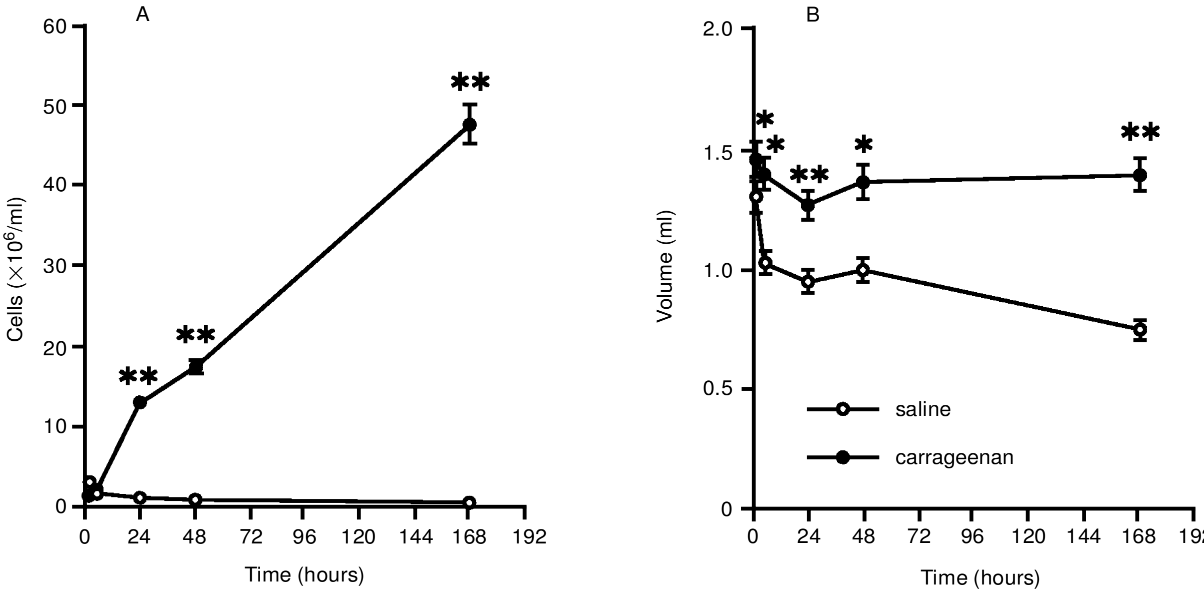

FIG. 1. Time course of total leukocyte number (panel A) and volume of exudate (panel B) in carrageenan-induced inflammation. Animals were pretreated with 1 ml of sterile saline (–○–) or 1 ml of 1% carrageenan in sterile saline (–●–). Data are the mean ± SD of at least eight animals. $^*P \leqslant 0.05$, $^{**}P \leqslant 0.01$ according to Duncan's test for multiple comparisons.

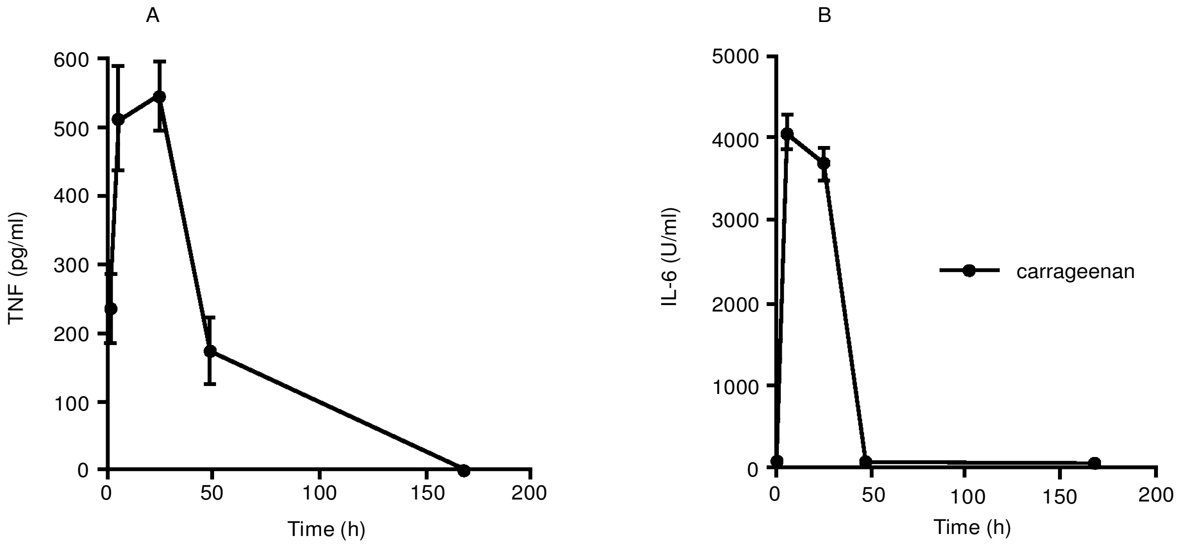

FIG. 2. Time course of TNF (panel A) and IL-6 (panel B) in carrageenan-induced inflammation. Animals were pretreated with 1 ml of sterile saline or 1 ml of 1% carrageenan in sterile saline (–●–). In saline-treated mice TNF and IL-6 were below the detection limit which was 50 pg/ml for TNF and 50 U/ml for IL-6. Data are the mean ± SD of duplicate determinations on at least eight animals.

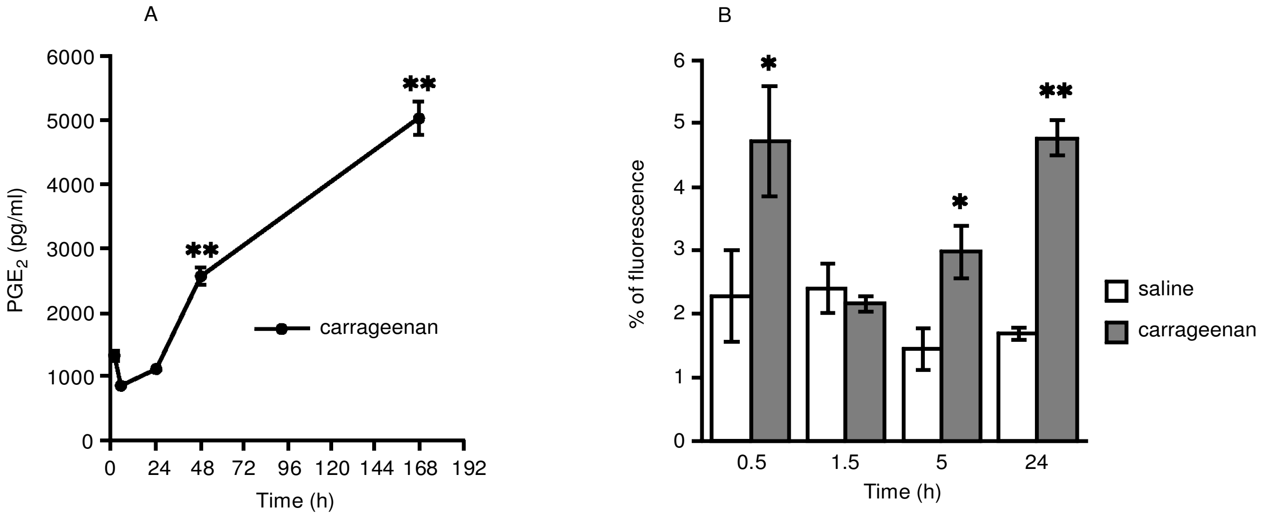

FIG. 3. Time course of $PGE_2$ (panel A) and plasma exudation (panel B) in carrageenan-induced inflammation. Animals were pretreated with 1 ml of sterile saline or 1 ml of 1% carrageenan in sterile saline. In saline-treated animals $PGE_2$ was below the detection limit which was 20 pg/ml. For the plasma exudation experiments FITC-BSA (0.6 mg/mouse dissolved in 0.2 ml/mouse of saline) was injected in the tail vein of animals 30 min before they were killed. Plasma exudation was expressed as a percentage of fluorescence in the pouch in relation to serum. Data are the mean ± SD of at least eight animals. *$P \leqslant 0.05$, **$P \leqslant 0.01$ according to Duncan's test for multiple comparisons.

### Effect of anti-inflammatory drugs on the various parameters of inflammation

The effects of systemic treatment with the anti-inflammatory drugs DEX or INDO are reported in Table 1. Both inhibited cell migration, the formation of exudate and the production of TNF, IL-6 and $PGE_2$. However DEX had much more effect than INDO.

### Effect of neutropenia

Neutropenia induced by pretreatment with 5-FU (Table 2), as expected reduced the number of leukocytes recruited into the pouch by carrageenan and the amount of TNF measured in the pouch. However the volume of exudate, plasma exudation and the concentration of $PGE_2$ were not different from the group treated with carrageenan alone.

**Table 1**. Effect of systemic treatment with dexamethasone or indomethacin on the inflammation induced by carrageenan in the mouse air pouch

| Treatment | Volume of exudate (ml) | Total leukocytes ($\times 10^6$/ml) | TNF (pg/ml) | IL-6 (U/ml) | $PGE_2$ (pg/ml) |
|---|---|---|---|---|---|
| Saline | 1.00 ± 0.001 | 1.31 ± 0.5 | ⩽ 50 | ⩽ 50 | 173 ± 23 |
| Carrageenan | 1.42 ± 0.08* | 28.95 ± 8.4* | 1470 ± 480** | ⩾ 6250** | ⩾ 3200** |
| Carrageenan + Dexamethasone | 1.02 ± 0.06 | 0.62 ± 0.19 | ⩽ 50 | ⩽ 50 | 983 ± 659 |
| Carrageenan + Indomethacin | 1.17 ± 0.08* | 13.8 ± 1.35* | 110 ± 40 | 3415 ± 2061* | 1170 ± 1523 |

One ml of sterile saline or 1 ml of 1% carrageenan in sterile saline were injected in 6 day-old pouches. 0.2% dexamethasone in drinking water was administered from 12 h before carrageenan. Two doses of indomethacin (2 mg/kg) were injected intraperitoneally 30 min before carrageenan and 8 h later. Animals were killed 24 h after the irritant. Data are the mean ± SD of at least five animals. $^{**}P \leqslant 0.01$, $^{*}P \leqslant 0.05$ according to Kruskal–Wallis and Sachs's test for multiple comparisons.

**Table 2**. Effect of 5-fluorouracil on the inflammation induced by carrageenan in the mouse air-pouch

| Treatment | Volume of exudate (ml) | Leukocytes ($\times 10^6$/ml) | Plasma exudation (% of fluorescence) | TNF (pg/ml) | $PGE_2$ (pg/ml) |
|---|---|---|---|---|---|
| Saline | 0.98 ± 0.07 | 2.88 ± 1.4 | 2.12 ± 0.67 | ⩽ 50 | ⩽ 10 |
| Carrageenan | 1.52 ± 0.08* | 16.4 ± 1.7** | 3.56 ± 1.27* | 487 ± 249** | 1640 ± 80** |
| 5-FU + Saline | 1.0 ± 0.05 | 0.61 ± 0.1 | 2.62 ± 0.5 | ⩽ 50 | ⩽ 10 |
| 5-FU + Carrageenan | 1.40 ± 0.17* | 2.1 ± 1.9 | 3.67 ± 0.89* | 101 ± 29 | 1500 ± 50** |

5-FU (150 mg/kg) was injected subcutaneously in the hind leg in 0.2 ml of $H_2O$ together with the first injection of air and a second dose was given on Day 6 when 1% carrageenan (1 ml) was injected in the pouch. Animals were killed 24 h later. Each value is the mean ± SD of at least eight different animals. $^{*}P < 0.05$, $^{**}P < 0.01$ according to Kruskal–Wallis and Sachs's test for multiple comparisons.

### Role of TNF

The protective effects of DEX and INDO, associated with an inhibitory effect on TNF production, suggest that TNF plays a role in this model of inflammation. To test this we used anti-TNF antibodies and IL-10, which inhibits TNF production. As shown in Table 3, anti-TNF antibodies (injected with carrageenan) reduced the induction of IL-6 in the exudate by 68% and cell recruitment by 44%

The dose of anti-TNF completely neutralized the levels of endogenous TNF induced by carrageenan (Table 3).

Reduction of inflammation associated with a lowering of TNF levels was confirmed by treating the animals with IL-10 (1 μg/mouse) administered into the pouch together with carrageenan or i.p. immediately before carrageenan. After 24 h the volume of exudate was not affected but TNF was inhibited by more than 90% in both experiments. Cell migration into the pouch was reduced by 67% and 58% respectively (data not shown).

Because mast cells were reported to be present in the lining of the pouch, we induced inflammation in a mast cell-deficient strain of mice to investigate the role of TNF in their granules. Table 4 reports a reduced inflammatory response 24 h after treatment. The inhibition of cell migration was particularly significant.

**Table 3**. Effect of anti-TNF antibodies on the inflammation induced by carrageenan in the mouse air pouch

| Treatment | Volume of exudate (ml) | Total leukocytes ($\times 10^6$/ml) | TNF (pg/ml) | IL-6 (U/ml) | $PGE_2$ (pg/ml) |
|---|---|---|---|---|---|
| Saline | 0.92 ± 0.12 | 1.18 ± 0.89 | ⩽ 50 | ⩽ 50 | 160 ± 33 |
| Carrageenan | 1.57 ± 0.17** | 15.47 ± 7.19** | 949 ± 542** | 3999 ± 3035* | 1150 ± 369* |
| Carrageenan + anti-TNF | 1.38 ± 0.18* | 8.72 ± 3.75**† | ⩽ 50†† | 1273 ± 796 | 1233 ± 251* |

Sterile saline (1 ml) or 1% carrageenan in sterile saline (1 ml) were injected in 6 day-old pouches. Anti-TNF antibodies (10 μl which diluted 1:12800 neutralizes 256 U of murine TNF) were injected into the pouches at time 0 together with carrageenan. The animals were killed 24 h after treatment. Data are the mean ± SD of at least eight animals in two different experiments. $^{*}P \leqslant 0.05$, $^{**}P \leqslant 0.01$ compared with the control group, $^{\dagger}P \leqslant 0.05$, $^{\dagger\dagger}P \leqslant 0.01$ compared with the carrageenan group according to Kruskal–Wallis and Sachs's test for multiple comparisons.

Table 4. Effect of mast-cell deficiency on carrageenan-induced inflammation in the mouse air pouch

| | Volume of exudate (ml) | Plasma exudation (% of fluorescence) | Total leukocytes ($\times 10^6$/ml) | TNF (pg/ml) | $PGE_2$ (pg/ml) |
|---|---|---|---|---|---|
| Controls | 1.46 ± 0.10 | 2.81 ± 0.53 | 30.8 ± 15.3 | 606 ± 306 | 1066 ± 472 |
| WBC6F1/J-Sl/Sl(d) | 1.45 ± 0.13 | 1.97 ± 0.91 | 17.9 ± 2.74* | 512 ± 480 | 850 ± 50 |

One ml/mouse of 1% carrageenan in sterile saline was injected in 6 day-old pouches of both experimental groups. The animals were killed 24 h later. FITC-BSA (0.6 mg/mouse) was injected i.v. 30 min before the death. Data are the mean ± SD of at least six animals. *$P \leq 0.05$ according to Student's t-test.

### Discussion

The objective of this study was to investigate the role of cytokines, particularly TNF, during the first phase of acute inflammation and to assess the relationship between these mediators and the time course of the cell migration and $PGE_2$ production. Other commonly used models of local inflammation, such as the subcutaneous injection of turpentine[21] or the carrageenan paw oedema,[22] give information on the oedematous response, but do not allow easy measurement of the cellular or humoral effects locally. The advantage of the air pouch model lies in its structural similarities with the synovial lining tissue[10] and the fact that the inflammatory exudate can be easily sampled and quantified for its cellular and biochemical composition. In this experimental model only a local inflammatory response is induced, with none of the systemic acute-phase changes usually observed in other models of local inflammation, including increases of IL-1, TNF, SAA, or hypoferraemia.[23] The confinement of the inflammation to the site of injection is not due to the particular structure of the pouch because TNF in serum was measurable when LPS, even at a low dose, was injected into the pouch. One possible explanation for this effect is that carrageenan is a less potent stimulus than LPS; it might also diffuse less than LPS.

The amount of TNF measured in the pouch seems to be related to the number of migrating cells. After treatment with 5-FU, when no cells are present in the pouch, TNF is below the detection limit. In addition the peak of TNF, 24 h after carrageenan, correlates with the increase of cells in the pouch. However the kinetics of cell infiltration is delayed compared with that of TNF and, since TNF is not present in serum, a possible source during the first phases might be the tissue of the pouch. In fact the lining of the pouch was reported to contain many mast cells,[24] the only cell type that stores TNF in the granules.[25] Therefore they might be the first source of TNF after stimulation and the TNF released from mast cells was reported to account for 40% of the PMN migration into the peritoneal cavity after immunocomplex-induced peritonitis and Arthus reaction in rats.[25] Using a mast cell-deficient strain of mice, carrageenan-induced cell migration was reduced 40%, seemingly in agreement with these data. However, the levels of TNF induced by carrageenan were comparable in normal and mast cell-deficient mice, indicating that these cells do not contribute significantly to TNF production in this model (evaluated 24 h after carrageenan). The protective effects of treatment with anti-TNF antibodies or IL-10 on some parameters of carrageenan-induced inflammation strengthen the suggestion that TNF plays a role in the process.

The time course of the production of $PGE_2$ parallels that obtained by Sin *et al.*[26] although the absolute amounts are completely different. We measured the $PGE_2$ released spontaneously in the exudate while Sin *et al.*[26] measured it both in cells and exudate. Since the increase of $PGE_2$ parallels the increase of cells in the pouch $PGE_2$ might be produced and released by leukocytes recruited and activated by carrageenan. However, after treatment with 5-FU, which induces neutropenia and prevents the accumulation of cells, the amount of $PGE_2$ was not affected, so it might also be formed by the pouch tissue and not by the migrating cells. These data seem in agreement with the ones in the rat reported by Sedgwick and Lees[27] and by Simmons *et al.*[28]

$PGE_2$ has been shown to down-regulate TNF production *in vitro*[12] and INDO, by breaking this negative feedback, potentiated LPS-induced TNF synthesis both *in vitro*[12,29] and *in vivo*.[30] We obtained contrasting results. Pretreatment with DEX or INDO reduced $PGE_2$ synthesis but also lowered TNF content. We measured TNF and $PGE_2$ after 24 h when the number of leukocytes in the pouch without INDO pretreatment was double that after INDO. Therefore if TNF is produced by leukocytes these data are not surprising.

Taken together the results indicate that TNF is a mediator of inflammation in the carrageenan-induced mouse air pouch model.

### References

1. Strieter RM, Kunkel SL, Bone RC. Role of tumor necrosis factor-α in disease states and inflammation. *Crit Care Med* 1993; **21**: S447–S463.
2. Remick DG. Significance of *in vivo* detection of tumor necrosis factor. *Lab Invest* 1991; **65**: 259–261.
3. Sekut L, Menius JA, Brackeen MF, Connolly KM. Evaluation of the significance of elevate levels of systemic and localized tumor necrosis factor in different animal models of inflammation. *J Lab Clin Med* 1994; **124**: 813–820.
4. Hopkins SJ, Meager A. Cytokines in synovial fluid: the presence of tumor necrosis factor and interferon. *Clin Exp Immunol* 1988; **73**: 88–92.
5. Husby G, Williams RC. Synovial localization of TNF in patients with rheumatoid arthritis. *J Autoimmun* 1988; **1**: 363–371.
6. Oliver TS, Noel LS, Stimpson SS, Yarnall DP, Connolly KM. Elevated levels of tumor necrosis factor in the joints of adjuvant arthritic rats. *Cytokine* 1993; **5**: 298–304.
7. Keffer J, Probert L, Cazlaris H, *et al.* Transgenic mice expressing human tumor necrosis factor: a predictive genetic model of arthritis. *EMBO J* 1991; **10**: 4025–4031.
8. Elliot MJ, Maini RN, Feldmann M, *et al.* Treatment of rheumatoid arthritis with chimeric monoclonal antibodies to tumor necrosis factor-α. *Arthritis and Rheumatism* 1993; **36**: 1681–1690.
9. Sedgwick AD, Sin YM, Edwards JCW, Willoughby DA. Increased inflammatory reactivity in newly formed lining tissue. *J Pathol* 1983; **141**: 483–495.
10. Edward JCW, Sedgwick AD, Willoughby DA. The formation of a structure with the features of synovial lining by subcutaneous injection of air: an *in vivo* tissue culture system. *J Pathol* 1981; **134**: 147–156.
11. Kunkel SL, Chensue SW. Arachidonic acid metabolites regulate interleukin-1 production. *Biochem Biophys Res Comm* 1985; **128**: 892–897.
12. Spengler RN, Spengler ML, Strieter RM, Remick DG, Larrick JW, Kunkel SL. Modulation of tumor necrosis factor-α gene expression. Desensitization of prostaglandin $E_2$-induced suppression. *J Immunol* 1989; **142**: 4346–4350.
13. Renz H, Gong JH, Schmidt A, Nain M, Gemsa D. Release of tumor necrosis factor-α from macrophages. Enhancement and suppression are dose-dependently regulated by prostaglandin $E_2$ and cyclic nucleotides. *J Immunol* 1988; **141**: 2388–2393.
14. Kushner I. The phenomenon of the acute phase response. *Ann NY Acad Sci* 1982; **389**: 39–48.
15. Gadina M, Bertini R, Mengozzi M, Zandalasini M, Mantovani A, Ghezzi P. Protective effect of chlorpromazine on endotoxin toxicity and TNF production in glucorticoid-resistant models of endotoxic shock. *J Exp Med* 1991; **173**: 1305–1310.
16. Catherine G, Bruyns C, Marchant A, *et al.* Interleukin-10 reduces the release of tumor necrosis factor and prevents the lethality in experimental endotoxemia. *J Exp Med* 1993; **177**: 547–550.
17. Howard M, Muchamuel T, Andrade S, Menon S. Interleukin-10 protects mice from lethal endotoxemia. *J Exp Med* 1993; **177**: 1205–1208.
18. Bentler B, Milsark IW, Cerami AC. Passive immunization against cachectin/tumor necrosis factor protects mice from lethal effect of endotoxin. *Science* 1985; **229**: 869–871.
19. Brennan FM, Chantry D, Jackson A, Maini R, Feldman M. Inhibitory effect of tumor necrosis factor-α antibodies on synovial cell interleukin-1 production in rheumatoid arthritis. *Lancet* 1989; **2**: 244–247.
20. Sipe JD, Gonnermann WA, Loose LD, Knapschoefer G, Xie WJ, Franzblon C. Direct binding enzyme linked immunosorbent assay (ELISA) for serum amyloid A (SAA). *J Immunol Methods* 1989; **125**: 125–135.
21. Won K, Campos SP, Baumann H. Experimental systems for studying hepatic acute phase response. In: Mackiewicz A, Kushner I, Baumann H eds. *Acute Phase Proteins*. Boca Raton, FL: CRC Press, 1993; 255–271.
22. Braga da Motta JI, Cunha FQ, Vargaftig BB, Ferreira SH. Drug modulation of antigen-induced paw oedema in guinea pigs: effects of lipolysaccharide, tumor necrosis factor and leucocyte depletion. *Br J Pharmacol* 1994: **112**: 111–116.
23. Fattori E, Cappelletti M, Costa P, *et al.* Defective inflammatory response in IL-6 deficient mice. *J Exp Med* 1994; **180**: 1243–1250.
24. Sin YM, Sedgwick AD, Chea EP, Willoughby DA. Mast cells in newly formed lining tissue during acute inflammation: a six day air pouch model in the mouse. *Ann Rheum Dis* 1986; **45**: 873–877.
25. Zhang Y, Ramos BF, Jakschik BA. Neutrophil recruitment by tumor necrosis factor from mast cells in immune complex peritonitis. *Science* 1992; **258**: 1957–1959.
26. Sin YM, Tan CH, Chan SF. Changes in histamine and prostaglandins in acute inflammatory air pouch in mice. *Agents Actions* 1989; **28**: 98–102.
27. Sedgwick AD, Lees P. Studies of eicosanoid production in the air pouch model of synovial inflammation. *Agents Actions* 1986; **18**: 429–438.
28. Simmons PM, Salmon JA, Moncada S. The release of leukotriene B4 during experimental inflammation. *Biochem Pharmacol* 1983; **32**: 1353–1359.
29. Watanabe S, Kobayashi T, Okuyama H. Regulation of lypopolysaccharide-induced tumor necrosis factor-α production by endogenous prostaglandin $E_2$ in rat resident and thioglycollate-elicited macrophages. *J Lipid Mediators Cell Signaling* 1994; **10**: 283–294.
30. Pettipher ER, Wimberly DJ. Cyclooxygenase inhibitors enhance tumor necrosis factor production and mortality in murine endotoxic shock. *Cytokine* 1994; **6**: 500–503.

**Received 13 September 1996; accepted in revised form 21 October 1996**
